# A rare combination of an endocrine tumour of the common bile duct and a follicular lymphoma of the ampulla of Vater: a case report and review of the literature

**DOI:** 10.1186/1477-7819-9-4

**Published:** 2011-01-14

**Authors:** Panagiotis G Athanasopoulos, Nikolaos Arkadopoulos, Vania Stafyla, Aliki Tympa, Evi Kairi, Charlotte Ryzman-Louloudis, Vassilios Smyrniotis

**Affiliations:** 1 Department of Surgery, University of Athens, Aretaieion Hospital, 76 Vas. Sofias Ave., 11528, Athens, Greece; 2 Department of Anesthesiology, University of Athens, Aretaieion Hospital, 76 Vas. Sofias Ave., 11528, Athens, Greece; 3 Department of Pathology, University of Athens, Aretaieion Hospital, 76 Vas. Sofias Ave., 11528, Athens, Greece; 4 4thDepartment of Surgery, University of Athens, Attikon Hospital, 1 Rimini Street, Chaidari 12462, Athens, Greece

## Abstract

Carcinoid tumours of the common bile duct represent an extremely rare entity. Similarly, primary follicular lymphomas of the ampulla of Vater constitute an infrequent neoplasia. Herein, we report the first case of a synchronous development of a carcinoid tumour of the common bile duct and an ampullary follicular lymphoma that was treated surgically with a Whipple's procedure, due to inability to establish definitive preoperative diagnosis despite the extensive diagnostic investigation.

## Background

Carcinoid tumours of the extrahepatic bile duct (EHBD) represent extremely rare lesions. These neoplasms account for 0.1% to 0.3% of all gastrointestinal carcinoid tumours [1]. Unlike cholangiocarcinomas, bile duct carcinoids occur more commonly in younger patients and in women [2]. Altogether, 70 patients with carcinoid tumours arising from the biliary tree have been reported in the literature [3]. Similarly, follicular lymphomas of the ampulla of Vater represent an infrequent entity accounting for only 1-3.8% of all gastrointestinal lymphomas [4] with only a few cases reported in the literature [4,5]. Interestingly, these cases most commonly occured in women. We report the first case of a synchronous carcinoid tumour of the EHBD and an ampullary follicular lymphoma that was treated surgically.

## Case Presentation

A 43-year old Caucasian male with no remarkable medical history, was referred to our clinic with intermittent jaundice and a 1-month history of nausea, vomiting, pruritus, dark urine, clay-colored stools and weight loss of 5 kg. The patient did not report abdominal pain or decreased appetite.

On admission, the patient's liver function tests (LFTs) were abnormal; aspartate aminotransferase (AST) 78 IU/L (normal, < 45 IU/L), alanine aminotransferase (ALT) 109 IU/L (normal, < 45 IU/L), alkaline phosphatase (ALP) 226 IU/L (normal, 40-150 IU/L), γ-glutamyl transpeptidase (γGT) 932 IU/L (normal, 10-55 IU/L), total bilirubin 1.3 mg/dl (normal, 0.21-1.2 mg/dl) and direct bilirubin 0.9 mg/dl (normal, < 0.5 mg/dl), while the tumour markers CEA, CA 19-9 and AFP were within normal range.

**Figure 1:**
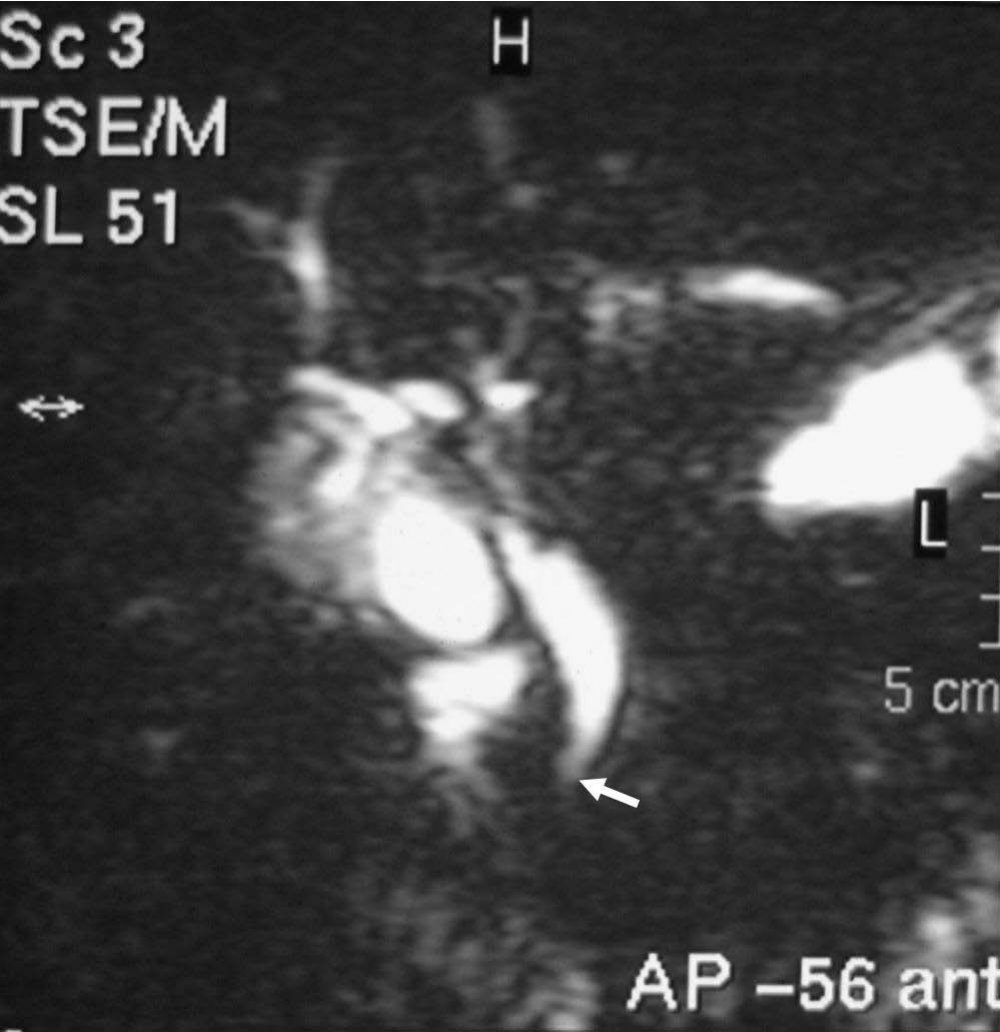
**MRCP depicting the dilatation and abrupt stenosis of the CBD, close to the ampulla of Vater (arrow)**.

Contrast enhanced computed tomography (CT) of the abdomen revealed dilatation of the biliary tree, with the hepatic duct measuring a diameter of 1.8 cm. Neither lymph node enlargement nor splenomegaly was demonstrated. Magnetic resonance cholangiopancreatography (MRCP) showed dilated intrahepatic and extrahepatic bile ducts including the cystic duct and the gallbladder. The common bile duct (CBD) measured 1.4 cm in diameter with an abrupt concentric stenosis in its lower third and a cut-off point located 1 cm distally to the duodenal ampulla. The pancreatic duct was depicted as normal (Figure 1).

Endoscopic ultrasonography (EUS) demonstrated the duodenal ampulla of Vater thickened and the CBD dilated with the presence of thick content.

The patient underwent an endoscopic retrograde cholangiopancreatography (ERCP). The ampulla of Vater was depicted as being infiltrated by the neoplasm. An endoscopic sphincterotomy was carried out, thus letting us better view and sample the region, and the biopsies taken revealed the presence of an undifferentiated neoplasm, but no definitive diagnosis was made.

Taking into consideration the aforementioned findings, our decision was to operate on the patient, and we performed a pylorus-preserving pancreaticoduodenectomy with the en bloc resection of the area in question and surrounding structures, including the regional lymph nodes.

**Figure 2:**
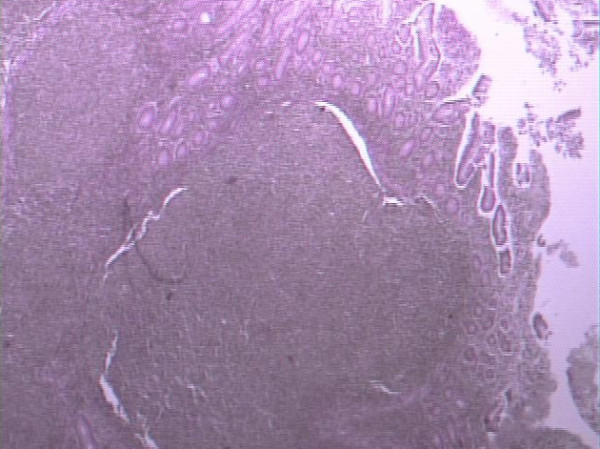
**Histological section of the duodenum showing a lymphoproliferative lesion consistent with a non-Hodgkin lymphoma (Hematoxylin & Eosin, ×250)**.

**Figure 3:**
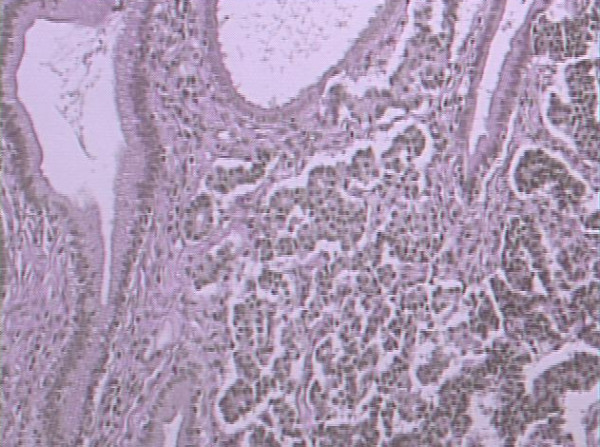
**Histological section of Vater's ampulla showing a carcinoid with insular pattern (H&E, ×250)**.

Histopathological and immunohistochemical study of the surgical specimen revealed infiltration of the duodenum by a non-Hodgkin B cell lymphoma, predominantly of nodular type, with characteristics of grade I follicular lymphoma [World Health Organization (WHO) classification] with G(λ)+ clonicity and indicative immunohistochemical examination (CD 10+, bcl-2-) (Figure 2). In addition, an intramucosal carcinoid of the CBD, measuring a diameter of 0.3 cm, was identified. All eight excised lymph nodes were normal (Figure 3).

Postoperatively, the patient was further staged. Regarding the lymphoma, we conducted a CT scan of the neck and chest as well as a bone marrow biopsy to exclude extra-enteric lesions, and we proceeded with a video capsule endoscopy in order to exclude multifocal involvement of the small intestine [6]. All these additional exams yielded no findings. As far as the carcinoid was concerned, the levels of the neuroendocrine markers 5-HIAA (5-hydroxyindoleacetic acid), CgA (chromogranin A) and NSE (neuron-specific enolase) were found within normal range. Because of the small size and histology of the carcinoid, no other imaging modalities such as Octreoscan or MIBG (meta-iodobenzylguanidine) scintigraphy were used [7].

The follow-up of the patient includes a complete blood count, routine biochemical exams and an abdominal CT scan every six months after surgery, supported with an annual evaluation of CgA levels [6,7]. Eighteen months after the resection, neither the lab tests nor the imaging techniques have revealed any recurrence of the disease nor has the patient referred any symptoms.

## Discussion

Carcinoid tumours arise from enterochromaffin cells, also known as argentaffin or Kulchitsky cells. The term enterochromaffin refers to the ability to stain with potassium chromate (chromaffin), a feature of cells that contain serotonin. These cells are located in the gastrointestinal tract (most commonly in the small intestine, appendix and rectum) [1] or at various sites within the respiratory tract [8] or the pelvic cavity (uterine cervix, ovary, testis), the oto-laryngeal region and the breast [9]. A small number of these cells also exist in the biliary tree [10]. The rarity of carcinoid tumours in this region are probably explained by the very limited number of the Kulchitsky cells [11]. Carcinoid tumours of the EHBD account for only 0.1-0.3 of all gastrointestinal carcinoids [1].

The term 'carcinoid tumour' has been replaced by the term 'well-differentiated endocrine tumour' in the latest WHO classification of tumours (IARC, Lyons, France, 2000). The malignancy of neuroendocrine tumours is well described, ranging from well-differentiated tumours to malignant neoplasms [1,12].

The WHO staging system is based on the criteria such as tumour size, histological differentiation, Ki-67 immunostaining, invasion of adjacent tissues, and vascular and perineural invasion.

Available data extrapolated from the existing literature suggest that carcinoid tumours of the extrahepatic biliary tree are of low malignancy [13]. However, these neoplasms tend to metastasize if remain untreated [9]. Complete resection of localized tumours, without metastases, results in a 5-year survival of 60-100% [1,14] thus proposing aggressive surgical management as an optimal treatment modality. In contrast, radiotherapy offers no benefit and chemotherapy has been used only in metastastic disease with too few reports to support any definitive conclusions [15].

Primary gastrointestinal non-Hodgkin lymphomas (NHL) constitute more than one-third of all extranodal lymphomas. The stomach is the most common site, followed by the colorectal region and the terminal ileum [16]. The duodenum is less frequently affected. The vast majority (80-90%) of primary gastrointestinal lymphomas originate from B cells and include diffuse large B cell lymphoma (DLBCL) and mucosa-associated lymphoid tissue (MALT) lymphoma, whereas follicular lymphomas have a reported incidence of 1-3.8% [4].

Compared to other biliary malignancies, lymphomas are more sensitive to chemotherapy and radiotherapy. However, NHLs represent a rare cause of malignant biliary obstruction, accounting for 1-2% of all cases [17], thus making medical decision-making a challenging procedure. Lymphomas of the periampullary region causing obstructive jaundice could be treated with chemotherapy provided that biliary drainage has been established securely through stenting of the CBD. Carcinoid tumours of the EHBD and ampullary follicular lymphomas, constitute exceedingly rare neoplasms. We herein report the first case of a synchronous development of these two malignancies that was treated surgically. Unlike most cases in the literature, where both carcinoids and follicular lymphomas exhibited a female predominance, our case refers to a 43-year-old male. Although the role of surgery for gastrointestinal lymphomas is not the treatment of choice, in our case resection was obligatory since the endoscopic biopsies were inconclusive.

## Conclusion

We present a patient harbouring a periampullary neoplasm, causing obstructive jaundice. We proceeded with a pancreaticoduodenectomy based on the preoperative finding of a poorly undifferentiated periampullary neoplasm. However, the final diagnosis revealed a follicular lymphoma of the duodenum and a carcinoid tumour of the CBD. No adjuvant therapy was judged appropriate after thorough staging and the patient is doing well eighteen months after surgery.

## Consent

Written informed consent was obtained from the patient for publication of this case report and any accompanying images. A copy of the written consent is available for review by the Editor-in-Chief of this journal.

## Competing interests

The authors declare that they have no competing interests.

## Authors' contributions

PA carried out the surgical procedure, designed the study, gathered the data and wrote the manuscript. NA finally revised the manuscript for submission. VSt participated in drafting and revising the manuscript. AT participated in gathering the data and drafted the manuscript. EK performed the appropriate histological analysis of the surgical specimens and provided histological sections as figures for the manuscript. CRL revised the language of the manuscript as a native English speaker. VS carried out the surgical procedure, participated in designing the study and revised the manuscript for submission. All authors have read and approved the final manuscript.
